# Community Knowledge and Acceptance of Larviciding for Malaria Control in a Rural District of East-Central Tanzania

**DOI:** 10.3390/ijerph110505137

**Published:** 2014-05-14

**Authors:** Leonard E. G. Mboera, Randall A. Kramer, Marie Lynn Miranda, Stella P. Kilima, Elizabeth H. Shayo, Adriane Lesser

**Affiliations:** 1 National Institute for Medical Research, 2448 Barack Obama Drive, P.O. Box 9653 Dar es Salaam, United Republic of Tanzania; E-Mails: stellakilima@yahoo.com (S.P.K.); eshayo@nimr.or.tz (E.H.S.); 2 Duke Global Health Institute, Duke University, 310 Trent Drive, Durham, NC 27710, USA; E-Mails: kramer@duke.edu (R.A.K.); adriane.lesser@duke.edu (A.L.); 3 Nicholas School of the Environment, Duke University, 9 Circuit Drive, Durham, NC 27708, USA; 4 School of Natural Resources and Environment, University of Michigan, 440 Church Street, Ann Arbor, MI 48109, USA; E-Mail: mlmirand@umich.edu

**Keywords:** malaria, larviciding, community acceptability, willingness to pay, Tanzania

## Abstract

The use of microbial larvicides, a form of larval source management, is a less commonly used malaria control intervention that nonetheless has significant potential as a component of an integrated vector management strategy. We evaluated community acceptability of larviciding in a rural district in east-central Tanzania using data from 962 household surveys, 12 focus group discussions, and 24 in-depth interviews. Most survey respondents trusted in the safety (73.1%) and efficacy of larviciding, both with regards to mosquito control (92.3%) and to reduce malaria infection risk (91.9%). Probing these perceptions using a Likert scale provides a more detailed picture. Focus group participants and key informants were also receptive to larviciding, but stressed the importance of sensitization before its implementation. Overall, 73.4% of survey respondents expressed a willingness to make a nominal household contribution to a larviciding program, a proportion which decreased as the proposed contribution increased. The lower-bound mean willingness to pay is estimated at 2,934 Tanzanian Shillings (approximately US$1.76) per three month period. We present a multivariate probit regression analysis examining factors associated with willingness to pay. Overall, our findings point to a receptive environment in a rural setting in Tanzania for the use of microbial larvicides in malaria control.

## 1. Introduction

Malaria is a major health challenge facing the developing world and sub-Saharan Africa in particular. In 2012, it was estimated that there were 627,000 malaria deaths worldwide, of which approximately 90% occurred in sub-Saharan Africa [1]. The use of microbial larvicides, a form of larval source management, is a less commonly used malaria control intervention that nonetheless has significant potential as a component of an integrated vector management (IVM) strategy [2]. In larviciding, mosquito larvae are targeted in their breeding habitats and are killed with an anti-larval agent. Historically, pesticides such as Paris Green were used, but this poses significant risks to humans, other non-target species, and the environment [3]. Modern preference for treatment of habitat is with the microbial agents of bacterial origin, *Bacillus thuringiensis* (Bti) and *B. sphaericus* (Bs) which attack the larvae of mosquitoes [4]. The effectiveness of microbial larvicides in reducing populations of mosquito larvae and adult mosquitoes in the surrounding area has been well-documented [5,6,7], but up to now the effect of larviciding on malaria incidence among humans is less clear and demands greater research. 

Microbial larviciding is an attractive malaria control intervention for a number of reasons. Both Bti and Bs appear to be safe; to date, neither Bti nor Bs have been shown to have any negative effects on non-targeted organisms, including humans [2,8,9]. Initial approximations suggest that larviciding is not only cost-effective, but also cost-competitive with other alternative malaria control strategies. Although data are sparse, one study estimates the cost of microbial larvicide protection per person per year to be between US$ 0.85 and 0.89 [7]. Compared with some other prominent alternative malaria control methods, such as insecticide treated mosquito nets (ITNs), the successful implementation of larviciding is less susceptible to issues with human behaviors such as uptake and consistent use. Moreover, because mosquito larvae cannot escape the bacteria in water, larviciding is not subject to the vector avoidance issue which has been raised as a concern with indoor residual spraying and ITN control methods [10]. 

The multiple potential benefits of larviciding reiterate the need for a multi-pronged IVM approach to malaria control. A package of malaria interventions addressing different stages and aspects of the disease and its management will have a greater impact. Both the IVM approach and literature on larviciding make clear that larviciding should never be a stand-alone approach, but rather explored as a promising complement to existing alternative malaria control methods. Larviciding has been shown to complement other malaria control methods. One study found that combining a microbial larviciding intervention with mass ITN distribution significantly improved control compared to mass ITN distribution alone [5]. As the evidence for larviciding as an effective non-chemical malaria control alternative builds, there is a heightened need to contextualize and define its place in the complicated array of malaria control methods. The absence of specific knowledge and capacity hinders the formulation of evidence-based national policy elements to promote and support larval source management in the early stage of the parasite life cycle. As an understudied intervention, the full role of larviciding as a malaria control measure remains to be clarified, especially in rural areas. In order for the full potential of larviciding to be realized, key stakeholders and decision-makers need more and clearer information on various parameters of its use, including its community acceptability.

Yet up to now, larviciding methods have remained understudied and undervalued [4], despite recognition and support from various sectors including national governments [11] and international organizations [9]. Existing studies on larviciding demonstrate the significant potential of larviciding but also highlight the immediate need for and value of greater research, particularly on innovative methods of larval source management [12,13,14]. Of particular relevance is a field study in rural Tanzania which found that two different types of microbial larvicide were safe, effective, and widely accepted by the community [6]. The study reported that the efficacy and persistence of the larvicides varied in different habitats and by larvicide type, underscoring the need to build a better understanding of the factors and contexts impacting the efficacy of different larviciding strategies. 

In particular, community-supported application of larvicide has the potential to be an innovative and sustainable method [13]. There has already been notable research establishing the feasibility of community-supported larviciding in an urban setting in Dar es Salaam, Tanzania [15], and the concept of enabling communities to implement and be engaged in local malaria control interventions has been promoted as a way to scale up IVM programming [16]. Yet the application of larviciding in rural areas remains understudied. One key element of determining feasibility of larviciding in a rural setting is community knowledge and acceptance of the intervention. Our objective was to assess community knowledge and acceptance of larviciding for malaria control in Mvomero District in east-central Tanzania as part of a larger community-supported larviciding experiment alongside a disease treatment intervention. Before implementing the experiments, this assessment provided the opportunity to evaluate baseline community acceptance, trust, and confidence in the effectiveness of larviciding, as well as reported willingness to pay (WTP) for a larviciding program. 

## 2. Experimental Section

### 2.1. Study Area and Sampling

This study took place in Mvomero district, located in east-central Tanzania. Mvomero district is situated in the Wami River Basin, alongside the Uluguru and Nguru Mountains. There are two rainy seasons in the area, occurring approximately from March–May and October–December. The area experiences high average annual rainfall (1,100 mm). The temperature in the area ranges from a mean minimum of 19 °C during the dry season (June–September), with October–March experiencing a mean maximum of 31 °C. Most adults (80%) are involved in agriculture. Malaria prevalence in the district was estimated at 34.5% in 2005 [17]. The most important malaria vectors in the district are *Anopheles gambiae* s.l. and *Anopheles funestus* [18]. Common water bodies in the study area vary by village but include rice paddies, ponds, puddles, road-side canals and ditches, streams, and temporary wetlands. 

This assessment took place during the baseline year of a larger cluster-randomized study evaluating a community-supported larviciding experiment alongside a disease treatment intervention. The study area consisted of 24 randomly-selected villages situated throughout six wards within the district. Within each village, approximately 40 households with at least one child under the age of five were randomly selected to participate in this study; a roster of eligible households (*i.e.*, with at least one child under the age of five) was compiled for each village based on local immunization registries and lists from an ITN government distribution program that targeted households with children under five. Randomization of villages to intervention groups did not occur until after baseline data collection was complete.

### 2.2. Data Collection

The survey instrument, focus group discussion (FGD) guide, and in-depth interview (IDI) guide were designed, based in part on our earlier surveys in 2005 [19] and 2007 [20]. The survey and focus group guide were piloted in Kibaha District and subsequently revised prior to full-scale data collection. All surveys, focus group discussions, and in-depth interviews were conducted in Kiswahili. 

#### 2.2.1. Quantitative Data

The structured questionnaire was administered to a head of the selected household, alternating between females and males whenever possible to maintain gender balance. With few exceptions, those approached agreed to participate in the baseline survey; these households comprised the baseline sample eligible to participate in subsequent longitudinal surveying during the study. The interviews sought information on the knowledge, attitudes, and perceptions of community members regarding malaria including transmission, risk factors related to malaria and larviciding. After initial questioning assessing participants’ baseline knowledge regarding larviciding, interviewers read to participants a standardized brief description of a proposed microbial larviciding program, including an assurance that “the substance is natural and safe for humans and animals” and that the program would “not put it in any drinking water”. Participants were then asked additional questions regarding their attitudes and perceptions regarding microbial larviciding. 

#### 2.2.2. Qualitative Data

Focus group discussions with community members and in-depth interviews with community leaders were conducted by research staff with professional experience and qualifications in the social sciences. Community leaders included village executive officers, village chairmen and village health workers. Facilitators used a uniform discussion guide during the in-depth interviews and focus group sessions which included discussion on awareness about malaria transmission and risk factors, mosquito population, and larviciding (acceptability, confidence in its ability to reduce the mosquito population and malaria incidence, and willingness to contribute to a larviciding program in one’s community). Each focus group consisted of participants of a single gender. A focus group was conducted in 12 of the 24 study villages, for a total of 12 focus groups (six male FGDs, and six female FGDs). The number of participants in each focus group ranged from 10 to 12. The discussions and interviews lasted roughly from one to two hours. Researchers conducted an in-depth interview in each of the study villages, for a total of 24 interviews. 

### 2.3. Ethical Considerations

This study was granted and maintained ethical clearance from the Medical Research Coordinating Committee of the National Institute for Medical Research in Tanzania as well from the Institutional Review Board of Duke University. Informed consent was obtained from survey, interview, and focus group participants. Confidentiality and privacy of participants was maintained throughout the study and study participants were assured of their rights to withdraw from the interview/discussion at any time.

### 2.4. Data Analysis

Qualitative data (in-depth interviews and focus group discussions) were transcribed by the same researchers who conducted the sessions, summarized key findings, and translated key representative quotations and dialogue into English. Household survey data were entered and evaluated for quality assurance by the study. Summary statistics and logistic regression models were conducted in STATA. Estimation of the lower-bound mean WTP was done in Excel. Results given are overall averages across all 24 study villages except where explicitly stated. The threshold for statistical significance was set at a *p*-value of < 0.05. 

## 3. Results and Discussion

### 3.1. Qualitative Results

Participants from FGDs and IDIs were generally aware that mosquitoes transmit malaria and understood the types of environments linked to an increase in mosquitoes, including mention of ponds and pools of water. About two-thirds of IDI informants understood that mosquito larvae were breeding in water bodies. Some informants specifically mentioned rice fields as contributing to larger mosquito populations. For instance, a village health worker commented, “*In our village there is a valley which contains water throughout the year, and people are practicing rice farming and hence creating habitats for mosquito breeding*”. This was also substantiated by the FGD participants: “*In this village we are not practicing intermittent irrigation so we usually grow rice in bunds that store water for a long time. Therefore this becomes a reservoir for mosquito breeding*” (FGD, male participant).

Despite the fact that the majority of participants from IDIs and FGDs were aware of malaria transmission and its contributing factors, they were generally unaware of larviciding as a malaria control method and had never seen it being used. Despite this lack of previous knowledge, after hearing a brief description of the method nearly all participants said they would support the introduction of larviciding in their communities. Feedback from focus group participants suggests two main reasons for their support are: (1) a basic understanding of how malaria is transmitted and how larviciding would logically interrupt the transmission cycle; and (2) the significant impact of malaria on health and finances in their households and communities. One male participant commented: “*Personally I think that I would agree on the introduction of larviciding because of two reasons. First, it kills mosquito larvae which could develop into adult mosquitoes and cause malaria. Second, we would save money which we are always spending to buy drugs for malaria treatment. When a member of the household falls sick there is a shift in budget allocation to buy drugs and there is a reduction of family income which could be used to assist in farming activities. A person can be kept for three weeks from participating in farming activities. In the absence of mosquitoes, I would not fall sick and could continue participating in production activities*”. The importance of larviciding was also echoed by the IDI participants. They said that because not everyone uses the mosquito nets properly, and many people stay outside of their homes until late at night, alternative measures could complement the use of nets: “*I think if we could have a program to kill mosquito larvae it will be good because even outside there will be no mosquitoes*” (IDI, Community leader). 

Based on the explanation provided about larviciding, a majority of participants thought that larviciding could reduce malaria incidence. In a women’s FGD group, there was widespread agreement from the group when one participant commented, “*I think malaria would be reduced significantly because mosquito larvae would be killed.*” In a different women’s FGD group, another participant similarly commented, “*the sources for mosquito breeding would be controlled and therefore there would be a reduction of adult mosquitoes and hence low malaria incidence.*”

Community leaders supported the introduction of a larviciding program and were confident that there would be no objection from community members. This statement was supported by community members participating in the FGDs. Despite the fact that participants showed a positive reaction towards larviciding, they insisted on community sensitization activities before its introduction. This would enable community members to understand its benefits and safety to humans, animals, and plants. For example, one community leader expressed during an IDI, “*Through community education, people will be able to understand the importance of larviciding and its safety.*” The same issue was raised by a FGD participant: “*If appropriate education were to be provided indicating larvicides do not harm animals and water, I think the community would accept the intervention*” (FGD, female participant)*.* A high level of trust towards larviciding was revealed as some of participants were even ready to volunteer to advocate to their neighbors the potential advantage of larviciding in their community. 

Indeed, focus group participants did express some concern and questions regarding where larvicide might be applied. In particular, some were worried about larvicide being applied to water bodies which are used as sources of drinking water or other domestic purposes. At the same time, participants were interested in additional details about the potency and duration of larvicide once applied and wondered whether heavy rains would wash away the product before it could take effect. 

Some participants expressed exaggerated expectations on the benefits of larviciding for malaria control, for example as revealed in the following quote: “*Through larviciding malaria would be reduced, if it is done every year especially during the rainy season there would be no mosquitoes and people would not suffer from malaria*” (IDI, community leader). Exaggerated expectations should be addressed during community education programming, especially since they could cause a risk that community members may be less likely to engage in other preventive methods or behaviors based on overestimation of the contributions of larviciding, as suggested by a female participant who commented that if a larviciding program were in place “*in case the mosquito nets are worn out there would be no need to repair them*”. However, after discussion participants seemed to understand the importance of using a combination of methods to combat malaria. 

Participants were asked about their willingness to contribute to sustain a microbial larviciding program. The majority of participants in the FGDs were willing to contribute a small amount of money to the program at regular intervals, e.g., 3 or 6 months with a minimum contribution of 1,000 Tanzanian Shillings (TShs.) (TShs. 1,000 is approximately US$ 0.60). One female participant said, “*Community members will be willing to contribute after seeing the way the larvae have been killed and the extent to which malaria has been reduced.*” Community leaders provided the same information on willingness to contribute but insisted it be an affordable amount (TShs. 1,000–3,000 per household). One of the FGD participants had this to say: “*People are saying that they would be willing to contribute but we could also refuse. I can provide an example. The fertilizers were brought to our village and we were supposed to pay TShs. 81,000. We loved to use fertilizer and we had trust in it. But in reality we couldn’t afford it. Using such an example, if this [larviciding] program is going to be introduced in our village the contribution rates should be in-line with our income level.*” A man in another FGD said, “*If we are informed that the chemical costs about TShs. 200,000 I don’t think we would be asked to contribute TShs. 100,000, rather we would contribute less. So the clarity on the amount to contribute would be provided by our experts based on the actual cost of the larvicide…Thereafter we would discuss and decide on the amount each household would contribute in order to get the required money.*”

Despite their willingness to contribute, some participants expressed reservations towards it because of concern of corruption and bad past experiences on community-supported programs. One had this to say: “…*in the past there were some women who visited our village and told us to contribute some amount of money for a water project. We did. But since then, we have never seen them, and nothing has been done*” (FGD male participant). Community leaders cautioned that community education would be required so that community members would be confident that the money they contribute would be used for its intended purposes. One village leader commented, “*I think people will agree to contribute because there are other programs which used to seek contributions from us so this will not be new. We make contributions for the construction of schools and dispensaries. What is needed is appropriate community sensitization.*” One leader linked the benefit of contribution and savings from malaria illnesses: “*After sensitization, the community will be able to contribute because an individual will not be happy to continue spending TShs. 5,000–10,000 for malaria treatment, while he or she can use TShs. 1,000 or 2,000 to eliminate mosquitoes*”*.*

Some FGD participants stated that community members’ willingness to pay (WTP) would be enhanced after observing positive results. A male FGD participant explained, “*I think if this program was introduced in our villages and we observe its impact we will trust it. If it will reduce the frequency of malaria we can buy [into] the project and contribute 100%.*” They also suggested that if the government were to initiate the program, community members would be more ready to contribute funds after seeing its benefits: “*If you will first implement this project and then ask us to contribute we would be willing. But if we are told to contribute before, for sure we won’t*” (FGD, male participant).

### 3.2. Quantitative Results

#### 3.2.1. Socio-Demographic Characteristic of Respondents

Table 1 shows demographic information for household survey respondents. A total of 962 heads of households were interviewed. The mean age of the respondents was 36.8 years. Males accounted for slightly more (54.0%) of the respondents. The mean number of individuals living in each household was 5.6 while the mean number of children under five per household was 1.3. Most (85.0%) of the respondents were living in a house that they or their family owned. The majority of the respondents owned a mobile phone (65.5%), radio (74.2%) and/or a bicycle (74.3%).

**Table 1:** Demographics of survey respondents.

Variable	Response	Value
**Sex**	Male	54.0%
	Female	46.0%
**Age (years)**	Mean	36.8 years (SD 11.9)
**Education level**	*Ever attended school:*	
	Yes	78.7%
	No	21.3%
	*Highest level attended:*	
	Pre-primary	0.5%
	Primary	89.4%
	Secondary	8.9%
	Higher	1.2%
**Household characteristics**	Mean number of people	5.6 people (SD 2.2)
	Mean number of children under 5	1.3 children (SD 0.7)
**Wealth Indicators - Own a:**	Motorcycle/scooter	8.8%
	Bicycle	74.3%
	Sewing machine	8.2%
	Radio	74.2%
	Television set	6.4%
	Sofa	19.7%
	Mobile phone	65.5%
**Rooms in compound**	1 room	8.6%
	2–3 rooms	61.0%
	4+ rooms	30.4%
**House/land ownership**	Owns house	85.0%
	Owns land	70.9%

#### 3.2.2. Knowledge, Attitudes, and Practices Regarding Malaria and Larviciding

Table 2 provides information on respondents’ knowledge, attitudes, and perceptions on malaria and vector control measures. Overall, household survey respondents demonstrated basic knowledge of malaria, including knowledge that mosquitoes transmit malaria (94.8%), knowledge of some factors affecting the mosquito population, and knowledge of actions that can be taken to reduce larvae and mosquitoes in their community. It was interesting to see that more than 60% of the respondents mentioned stagnant water as a factor related to both mosquito and larvae populations. However, 23.3% reported that they did not know where larvae are found. Overall, respondents were also knowledgeable of actions that can be taken to reduce larvae and mosquitoes in their community. For instance, 35.4% and 43.1% of the respondents indicated that draining stagnant water around the home was a way to reduce mosquitoes and mosquito larvae in the community, respectively.

**Table 2:** Knowledge, attitudes, and perceptions on malaria and vector control.

Variable	Percentage
General Malaria Knowledge	
Knowledge that mosquitoes transmit malaria	94.8%
Perception that there are mosquitoes in the respondent’s community	99.1
Knowledge of factors affecting mosquito population	
Rainfall	21.2
Stagnant water	67.8
Cleanliness of village/surroundings	67.5
Humidity	6.5
Knowledge of where mosquito larvae are found	
Stagnant water/irrigation ditches	69.9
Forest	15.3
Pit latrine	7.7
Vegetation around the house	13.7
Don’t know	23.3
Malaria Vector Control	
Knowledge that reducing mosquito population can help reduce malaria	90.0
Mention of actions that can reduce mosquito abundance	
Drain stagnant water	35.4
Clearing grass/bushes around home	28.1
Spray insecticides	7.7
Use larvicides	8.4
Clean environment around home	59.9
Use bed nets	50.3
Mention of actions that can reduce mosquito larvae abundance	
Drain stagnant water around home	43.1
Clearing grass/bushes around home	18.8
Use larvicides	16.7
Clean environment around home	41.4
Don’t Know	25.9

Table 3 contains survey respondents’ attitudes towards larviciding for malaria control. A small proportion of respondents (17.8%) reported having knowledge of larviciding. After the interviewer read the standardized brief description of microbial larviciding, including an assurance that “the substance is natural and safe for humans and animals” and that the program would “not put it in any drinking water”, respondents were asked whether they trusted that the larvicide would not cause harm to humans and livestock. A majority (73.1%) posed with this dichotomous question said they did trust in the safety of larvicide as it had been described to them. All respondents were asked whether they would give permission for the project to conduct larviciding in water bodies near their homes where mosquitoes breed; 92.9% said yes. The vast majority (92.3%) said they had confidence that larviciding would reduce the number of mosquitoes around their home, and a similar percent (91.9%) said they would have confidence that larviciding would reduce the risk of someone in their household getting malaria.

**Table 3:** Household attitudes towards larviciding: Dichotomous responses.

Variable	Percentage Responding Yes
Knowledge of larviciding	17.8
Trust in safety of larvicide	73.1
Permission for program to apply larvicide in bodies of water near home	92.9
Confidence that larviciding will reduce number of mosquitoes	92.3
Confidence that larviciding will reduce risk of getting malaria	91.9

Those who answered yes to the questions regarding larvicide safety and efficacy were queried further to ascertain their level of confidence (*i.e.*, the likelihood of each component being true) using a 5-point Likert scale. This approach revealed additional details about confidence levels of those answering positively. The results are presented in Table 4. Of those that said they trusted in the safety of larvicide, only 20.4% said they thought it was highly likely that the larvicide was safe (*i.e.*, high level of confidence in its safety), with 46.4% reporting it was likely that larvicide was safe. Among those that said they had confidence that larviciding would reduce the number of mosquitoes around their home, 18.2% felt it was very likely, while 46.7% thought it was likely. While only 15.7% thought it very likely that larviciding would reduce the risk of getting malaria, about half (49.2%) thought it likely.

**Table 4:** Likert scale responses to questions regarding attitudes towards larvicide.

Response	Very Likely	Likely	Neutral	Not Likely	Very Unlikely
Likelihood that larvicide is safe for humans and animals	20.4%	46.4%	26.4%	6.8%	0.0%
Likelihood that larviciding will reduce number of mosquitoes	18.2%	46.7%	26.8%	8.2%	0.1%
Likelihood that larviciding will reduce risk of malaria infection	15.7%	49.2%	26.8%	8.3%	0.0%

#### 3.2.3. Willingness to Pay for a Larviciding Program

Willingness to pay is described as the maximum a person or household would be willing to pay for a good or service. It is one approach for providing an estimate of benefit for use in cost benefit analysis [21]. Survey participants were asked whether they would be willing to contribute a specific amount every three months for a larviciding program to be implemented in their community. The contribution amount was randomly prepopulated in each survey from among the possibilities of TShs. 1,000, 2,000, 3,000, or 4,000. The payment amounts were randomly pre-populated into the surveys before they were administered, with each payment amount included in an equal number of surveys (*i.e.*, the probability of any of the payment amounts being specified was nearly equal across all respondents).

**Figure 1:**
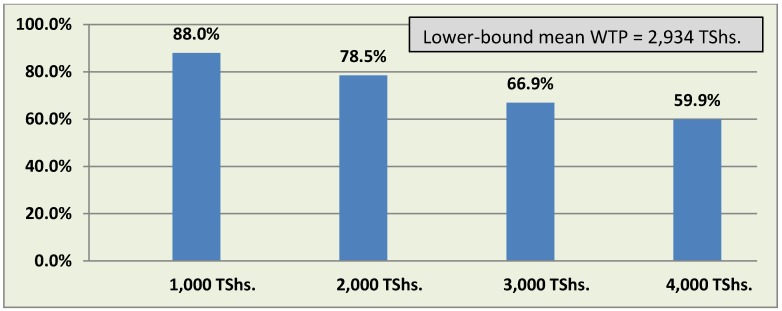
Willingness to pay for a larviciding program, by contribution amount per three-month period (%).

Overall, household survey respondents indicated a significant willingness to pay for a larvicide program in one’s community, with 73.4% overall responding that they would be willing to contribute across all possible contribution amount queries. As expected, there was a decrease in WTP as the contribution amount increased; 88.0% of respondents asked whether they were willing to contribute TShs. 1,000 every three months responded affirmatively, while this dropped to 78.5% for the amount of TShs. 2,000, 66.9% for the amount of TShs. 3,000, and 59.9% for the amount of TShs. 4,000 every three months (Figure 1). 

The mean WTP was calculated using a nonparametric maximum likelihood method initially proposed by Turnbull [22] and further developed by Carson *et al.* [23]. An application of this approach yielded a lower-bound mean WTP of 2,934 TShs. per three month period.

#### 3.2.4. Regression Analysis

In this section, we examine socioeconomic and attitudinal factors associated with respondents’ willingness to pay for a larviciding program for malaria control in their communities. Below we present a model that explores how socioeconomic characteristics and attitudes related to malaria and larviciding are related to respondents’ expressed WTP (Table 5).

Table 5 shows the results of a multivariate probit analysis evaluating the association of demographic, socio-economic status (SES), self-reported malaria, and attitudinal factors with willingness to make a recurring contribution to a proposed larviciding program. Among demographic factors, whether a respondent had ever attended school was significantly associated with WTP in a positive direction. Living in a house that had an improved roof (constructed of iron sheets rather than grass, leaves, or mud) is an indicator of household wealth in the study area; this factor also had a statistically significant positive association with WTP. Respondents expressing trust in the safety of larviciding (posed as a dichotomous question) were also more likely to be willing to pay. Finally, as would be expected, there was a strong negative association between the bid amount (*i.e.*, the proposed contribution) and WTP. 

**Table 5:** Multivariate probit analysis of the associations between socioeconomic, self-reported malaria, and attitudinal indicators with expressed willingness to pay.

Variable Type	Variable	Coefficient	95% CI	z	*p*-Value
**Demographics**	Respondent age	−0.003	−0.012–0.005	−0.78	0.434
	Respondent ever attended school	0.449	0.213–0.685	3.73	0.000 *
	Number of people in the household	0.027	−0.020–0.075	1.12	0.262
	Respondent’s main occupation–crop farming	−0.122	−0.391–0.148	−0.88	0.377
**Wealth**	Improved roof (iron sheets instead of grass/leaves/mud)	0.309	0.104–0.514	2.96	0.003 *
**Reported malaria**	≥1 recent case of self-reported malaria in household (assessment of each member’s most recent fever if within past 3 months)	−0.048	−0.252–0.155	−0.47	0.641
**Attitudes towards larviciding**	Trust in safety of larviciding	0.350	0.107–0.593	2.82	0.005 *
**Bid**	Larviciding contribution amount	−0.340	−0.429–−0.250	−7.42	0.000 *

Notes: Willingness to Pay (Y/N); N = 792; Prob > chi^2^ = 0.000. * Significant at the 0.05 level.

The number of people in the respondent’s household and the indicator variable for perceived burden of malaria in the household were included in the model as representations of the respondent’s perceived level of risk of malaria infection in their household, a perception which one could assume would be higher when there was a greater number of people at risk in the household, and when someone in the household was perceived to have had malaria recently. A greater perception of malaria infection risk to household members could affect the perceived benefits of, and thus WTP for, a larviciding program targeting malaria vector control. However, neither the number of people in the household nor self-report of a recent case of malaria in the household had a statistically significant association with WTP. There was also no statistically significant association with WTP for the variables of respondent’s age or occupation (whether or not the respondent was a crop farmer).

### 3.3. Discussion

Generally, knowledge of malaria transmission, breeding sites and prevention measures was good among communities of Mvomero District. Previous studies in the area have shown a similar picture, though most of the people could not associate human activities such as farming and malaria transmission factors [19]. Most respondents believed that the majority of fever episodes experienced by household members were due to malaria. 

Less than half of the respondents had knowledge of the usefulness of larval source management in the control of malaria. The majority of people in Mvomero district were unaware of larviciding as a malaria control method. Low community knowledge on larval source management was expected. In Tanzania, during the past decade, larviciding for malaria control has been concentrated in the City of Dar es Salaam [13,14,24,25]. In Tanzania [26], like elsewhere in Sub-Saharan Africa, the use of larval source management as a strategy for malaria control has received little attention from both policy makers and implementers despite historical success elsewhere [27]. This is likely to have contributed to low knowledge of the intervention among people in the country. However, since larviciding trials were carried out in some villages of the Mvomero study area during 2006–2008 [6], a larger proportion of respondents having some knowledge of this approach was expected. The lack of familiarity with larviciding among the respondents is likely related to the low level of community involvement in the previous trial. 

Questions arose in the focus groups about the effectiveness of larviciding. Studies in the urban area of Dar es Salaam have shown that regular larviciding had a significant protective effect on malaria prevalence [25]. In fact, larviciding showed synergistic effects with the use of insecticide-treated mosquito nets and house proofing [25]. However, such success in an urban setting may not be realized in a rural setting because of the differences in nature of the breeding sites. Most often, larval habitats are less numerous and easier to access in an urban setting than in a rural setting. Interestingly, the observation by Maheu-Giroux and Castro [25] that larviciding during the dry season was more effective at lowering the prevalence of malaria infection than during the rainy season may be utilized in a rural setting as a basis for application of larvicides just before the rainy season starts. When rainfall is low, larviciding activities are likely to be more effective at suppressing larval production due to operational issues [25].

Perceptions regarding the safety of larviciding were mentioned as an important challenge in the acceptability of the intervention. Studies elsewhere have shown that microbial larvicides are environmentally safe, specific in their action, and highly effective in killing *Anopheles* larvae under field conditions [2,6,28,29]. Eliciting survey respondents’ level of trust in the safety of larviciding through a 5-point Likert scale demonstrates that even though the majority of respondents said they trust in the safety of larviciding, their level of confidence may not be high (which is understandable particularly given that the vast majority was previously unaware of larviciding). These results support the dialogues in the focus group and in-depth interview discussions which stressed additional community sensitization activities would be necessary before introduction of a larviciding program to bolster community acceptance and trust. A large majority of respondents said they would give permission despite the relatively small proportion of respondents reporting the highest level of trust in its safety. Respondents might feel that despite a lack of confidence in the safety of larvicide, the threat of malaria is greater and consenting to larviciding near their homes is therefore worth the perceived risk or trade-off.

Interestingly, despite the low awareness of larviciding among the respondents, a very high level of interest and confidence in the effectiveness of larviciding was experienced by the majority of the people surveyed. In fact, as noted, the effectiveness of larviciding particularly in a rural setting is an understudied area in the scientific literature, and while its effectiveness in reducing larvae (and thereby mosquito population) has been proven through many studies, less is known about the extent of its ultimate impact on actual malaria incidence. A study in the same district by Magesa *et al.* [6] has shown that microbial larvicides are effective in controlling mosquito productivity. However, in their study, they did not measure malaria parasitemia prevalence among the residents of the villages under the trial. The high level of interest and confidence in the effectiveness of larviciding shown in this study provide an opportunity to introduce community-based larval source management interventions. In the neighboring district of Kilosa, it has been reported that a proportion of respondents stay outdoors late at night, reducing the utility of nets in protecting against mosquito bites [30]. Nighttime outdoor activities reported by the respondents have implications for malaria transmission and control. Some members of the *Anopheles gambiae* complex are known to readily seek hosts outdoors [31]. 

In this study, the willingness to pay for a larviciding program was also explored in the FGDs and the household survey. Studies on willingness to pay for malaria control interventions in Sub-Saharan Africa are few [32]. The findings of our study indicate that there is high level of WTP (demand) for larviciding. Overall, household respondents expressed a willingness to pay for a larvicide program, with three quarters of the respondents willing to contribute to a larvicide program across all contribution amounts, but with a decrease in willingness to pay as the contribution amount increased as expected. The mean WTP was estimated at TShs. 2,934 (or approximately US$1.76) per three month period. The multivariate probit regression analysis found statistically significant associations between WTP and variables indicating trust in the safety of larviciding, education, and wealth. Previously in Sudan, WTP for malaria control interventions was found to depend on the socio-economic status of the individual [33]. In Sudan, comparing three malaria control interventions, Onwujekwe *et al.* [33] observed that ITNs had the highest mean WTP followed by indoor residual house-spraying, while larviciding had the least. In our study, it was found that most participants were willing to contribute a small amount of money to support a larviciding program. In community-based health interventions, the determination of consumer preferences and demand is important, when viewed in the context of community involvement and the need for local support for financial sustainability of malaria control [33].

Successful community-based integrated malaria vector control programs in both rural and urban settings have been reported in Kenya and Tanzania, respectively [16]. In both programs, larval source management was part and parcel of the interventions. As reported in this study, community mobilization through appropriate sensitization was a key component in the two programs [16]. As previously described by Rogers and Shoemaker [34], the dissemination of information and adoption of appropriate malaria control activities require all the elements of the classic theory of the Diffusion of Innovations. This means that community-based malaria mosquito larval control should be communicated through personal interaction during community training sessions focused on learning-by-doing [16].

There are several limitations to this study. Individuals queried about their level of trust or confidence in the effectiveness of larvicide (a potential benefit to the community) might have felt pressure to express support for such a program when surrounded by peers (as in FGDs) or in a leadership position (as with IDIs). Similarly, survey responses may have been influenced by the presence of interviewers from outside the local communities. Among those who responded that they did trust in the safety and/or efficacy of the proposed larviciding program, probing further using a 5-point Likert scale allowed for a greater understanding with regards to their level of trust. Willingness to pay responses may have been biased by the hypothetical nature of the questions since respondents may not have expected to actually have to pay at some future time. 

## 4. Conclusions

This study indicates that rural community members were likely to be receptive to larviciding for malaria control in east-central Tanzania, more so following adequate community sensitization. Members of the households were willing to contribute across a range of possible quarterly payment levels to support a larviciding program in their villages. In conclusion, the results of this study indicate a receptive environment for future efforts directed at larviciding for malaria control in a rural setting in Tanzania.
